# Regenerative Medicine and the Developing World

**DOI:** 10.1371/journal.pmed.0030381

**Published:** 2006-09-12

**Authors:** Heather L Greenwood, Peter A Singer, Gregory P Downey, Douglas K Martin, Halla Thorsteinsdóttir, Abdallah S Daar

## Abstract

This is the first study to systematically identify and prioritize which applications of regenerative medicine are the most promising for improving health in developing countries.

A panel of experts at the annual meeting of the American Association for the Advancement of Science has predicted that stem cell research will survive the fallout of the recent scandal involving falsification of data by South Korean stem cell researcher Woo Suk Hwang [1]. As the field of regenerative medicine regroups, it is important that recent events do not overshadow the potential benefits of the field. These benefits include the opportunity to improve health care for the more than two-thirds of the world's population who live in developing countries.

While developing countries face pressing priorities in providing basic health services to their populations, they are also increasingly struggling to manage epidemic rates of noncommunicable diseases whose prolonged and costly care is drawing significant resources away from these basic priorities [2–4]. Although developing countries have been largely neglected by the field of regenerative medicine to date, we suggest that they could potentially benefit from advances in regenerative medicine to address the epidemic of noncommunicable diseases and other pressing health needs.

Regenerative medicine is an emerging field that seeks to combine the knowledge and expertise of diverse disciplines towards the aim of healing impaired function in the body [5,6]. Its goal is not just to replace what is malfunctioning, but to provide the elements required for in vivo repair, to devise replacements that seamlessly interact with the living body, and to stimulate the body's intrinsic capacities for regeneration [7]. The United States National Academies of Science report, *Stem Cells and the Future of Regenerative Medicine*, estimates that the potential patient populations in the US for stem cell–based therapies include more than a hundred million patients with conditions such as cardiovascular disease, autoimmune diseases, diabetes, cancer, neurodegenerative diseases, and burns [8].

Though still an emerging field, regenerative medicine has already produced a number of therapies, including the tissue-engineered skin substitute Apligraf [9] and the adult stem cell–containing bone regenerating therapy Osteocel [10]. The L. V. Prasad Eye Institute in India has treated blindness in more than 125 patients using adult stem cell therapy for corneal repair [11]. Ninety percent of those affected by blindness live in poor communities and it is estimated that 60 percent of conditions that cause blindness are treatable [12]. Regenerative medicine could

However, despite the fact that the conditions targeted by regenerative medicine are more prevalent in developing than developed countries [2,4,13,14], there has been no attempt to systematically understand how regenerative medicine could contribute to improving health in developing countries. Eighty percent of the world's chronic disease deaths [2], more than 95 percent of infectious disease deaths [13], and almost 90 percent of deaths due to injury and trauma [4,14] are found in low- and middle-income countries. Chronic diseases, the primary targets of regenerative medicine, affect people at a younger age in developing than developed countries, are much more likely to occur in the poor than the rich in all but the least-developed countries, and resulted in more deaths in 2005 than infectious diseases, maternal and perinatal conditions, and nutritional deficiencies combined [2].

This paper discusses some of the ways in which regenerative medicine could potentially be used to meet the health needs of developing countries. We do not suggest that regenerative medicine presents easy solutions to the complex challenges facing developing countries, but encourage researchers and policy makers in both developing and developed countries to consider how this new technology might address the health needs of the developing world. First, we present results of a study that systematically identifies and prioritizes applications of regenerative medicine that could potentially be effective in improving health in developing countries. Second, we discuss the feasibility of building capacity in regenerative medicine in developing countries. Finally, we discuss implications and provide recommendations for both developed and developing countries.

## Top Ten Regenerative Medicine Applications for Improving Health in Developing Countries

**Figure 1:**
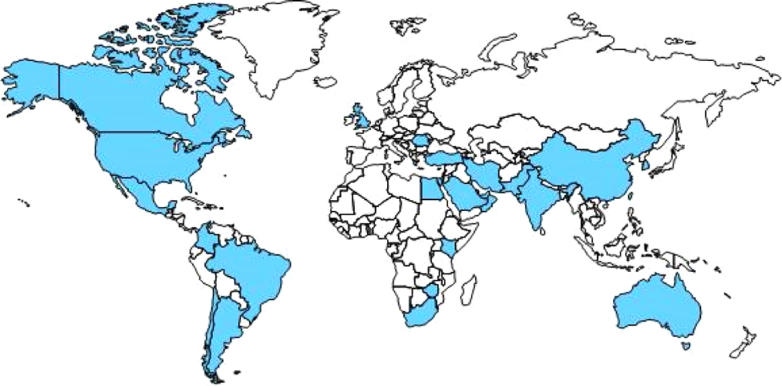
Countries Represented on the Expert Panel potentially provide more affordable treatments than corneal grafts and could address current shortages of donor material.

An international panel of 44 experts, including researchers in disciplines contributing to regenerative medicine and clinicians working in fields that will be at the forefront of applying regenerative medicine therapies, participated in a technology foresight study to identify the ten most promising applications of regenerative medicine for improving health in developing countries (Table S1). We made a conscious effort to balance specialty areas within regenerative medicine, geographic distribution (see Figure 1), and gender representation on the expert panel. Seventy-seven percent of the panellists lived in developing countries as defined in the United Nations Human Development Report 2005 [15].

**Definition of Regenerative Medicine Used in Our Study**

Regenerative medicine is an emerging interdisciplinary field of research and clinical applications focused on the repair, replacement, or regeneration of cells, tissues, or organs to restore impaired function resulting from any cause, including congenital defects, disease, and trauma. It uses a combination of several technological approaches that moves it beyond traditional transplantation and replacement therapies. These approaches may include, but are not limited to, the use of stem cells, soluble molecules, genetic engineering, tissue engineering, and advanced cell therapy.

A modified Delphi method, as described in two previous studies [16,17], was used to build consensus among the experts through a series of three rounds. Communication with the panellists occurred primarily via e-mail, while phone and fax served as supporting and alternative means of contact. In the first round, panellists were asked the open-ended question, “What do you think are the clinical applications of regenerative medicine that are the most likely to improve the health of people in developing countries within the next ten years?” We provided the definition of regenerative medicine that is shown in the sidebar.

This definition was developed based on a previously published definition [6], which was modified and validated based on input from regenerative medicine experts external to the panel. In response to the open-ended question, panellists proposed applications and provided comments to support their suggestions. Results were analyzed and grouped according to common themes, while suggestions that did not fit within the definition of regenerative medicine were omitted. The resulting list was reviewed for face validity by two experts in regenerative medicine external to the panel.

In the second round, panellists were asked to rank their top ten choices from the list of 29 applications derived from the first round. The rankings of each panellist were added together to provide a cumulative score for each application. In order to provide an increased number of choices and more accurate results, the top 14 applications were chosen to redistribute to the panellists for the third round along with a brief summary of the panellists' reasons supporting each choice. Consensus was consolidated as panellists were asked to either agree with the ranking or to re-rank their top ten choices from the list of 14. In addition, concrete examples were gathered from the panellists in the third round. The panellists' comments were used to identify six criteria that informed their choices (Box 1).

**Figure 2:**
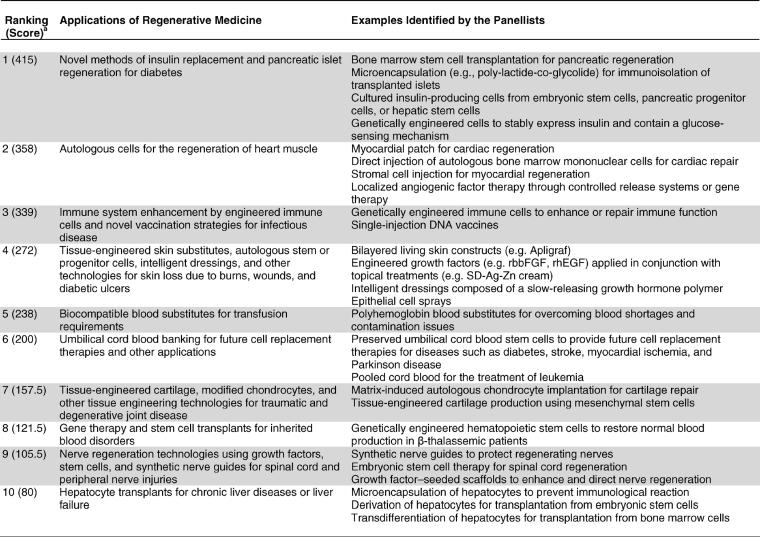
Top Ten Regenerative Medicine Applications for Improving Health in Developing Countries ^a^The maximum total score an application could receive was 440.

Figure 2 contains the list of the ten regenerative medicine applications considered by the panellists to be the most promising for improving health in developing countries. There was a high degree of consensus with regard to the top five applications: all panellists except one ranked at least three of the top five applications in their own top five responses. The number one ranked application, “Novel methods of insulin replacement and pancreatic islet cell regeneration for diabetes” received 415 points out of a possible maximum of 440 (44 × 10), and was ranked in first position by 35 of the 44 panellists. Due to space restrictions, only the top three applications are discussed in further detail below.

### Top-ranked application: Novel methods of insulin replacement and pancreatic islet cell regeneration for diabetes.

The first-ranked application was frequently supported by reference to the high prevalence of diabetes in developing countries and the major health, social, and economic burden that results. Panellists emphasized that controlling diabetes would in turn reduce the incidence of complications such as blindness, heart disease, chronic kidney disease, and diabetic ulcers. The panellists noted that repeated insulin treatments are costly and inaccessible to many patients in developing countries. They felt that regenerative medicine therapies, such as bone marrow stem cell transplantation or microencapsulated islet cells using novel biomaterials, could increase accessibility by providing a permanent solution and reducing the financial burden caused by the purchase of insulin.

### Second-ranked application: Autologous cells for the regeneration of heart muscle after myocardial infarction and cardiomyopathies.

Dramatically increasing rates of cardiovascular diseases in developing countries were cited by panellists in support of the second-ranked application. Panellists noted that such therapy shows promising early results, and is being tested in clinical trials in a number of countries. Panellists believed that in addition to saving lives, such therapies could potentially reduce the cost of treating heart failure. Autologous cells, potentially injected directly into damaged regions of the heart or used in regenerative myocardial patches, were emphasized by the panellists due to their advantage of avoiding immune rejection and, hence, costly immunosuppressive regimens.

### Third-ranked application: Immune system enhancement by engineered immune cells and novel vaccination strategies for infectious disease.

In support of this application, panellists referred to the devastating burden of infectious diseases in developing countries, particularly on the young. According to the panellists, the regeneration or enhancement of the immune system by engineered immune cells and novel vaccination strategies could improve an individual's ability to fight infections and to combat new strains of common diseases. Panellists made specific reference to the importance of addressing HIV/AIDS, tuberculosis, hepatitis, and malaria.

## Feasibility of Building Capacity in Regenerative Medicine in Developing Countries

This study shows that regenerative medicine could potentially be applied towards improving health in developing countries. The results highlight regenerative medicine applications that could be relevant for addressing the growing epidemic of chronic diseases in the developing world. Some of these chronic diseases, such as diabetes and cardiovascular disease, are receiving significant research attention from industrialized nations. In addition, this study highlighted applications relevant to addressing the persisting burden of infectious diseases in developing countries. These diseases generally do not receive as much attention from industrialized nations. Applications relevant to infectious diseases include therapies for immune system enhancement and biocompatible blood substitutes that can be sterilized to avoid costly screening measures.

Whether developing countries choose to build capacity in regenerative medicine themselves or whether they wait to adopt therapies developed first elsewhere may depend both on their economic position and on the level of research attention a particular regenerative medicine application receives in industrialized nations. However, as highlighted in the 2005 report of the United Nations Task Force on Science, Technology, and Innovation, domestic innovation by developing countries is important because it is more likely to be targeted towards local health needs and can contribute to health and economic development [18]. In addition, previous studies of the health biotechnology sectors in developing countries have shown that local innovation in science and technology can lead to more affordable treatments for the populations of developing countries [19]. India's Shantha Biotechnics, for instance, has developed a recombinant hepatitis B vaccine that sells for only $US0.40 per dose as compared with imported vaccines that sell for $US8–$US10 per dose [20].

The ability of developing countries to build capacity in regenerative medicine is shown by activities already underway in a number of developing countries. As may be expected given the economic variance among developing countries, India, China, and Brazil currently show higher levels of regenerative medicine activity than less-developed countries [7].

India's federal government, for instance, has launched a national stem cell research program that will establish six research clusters in six cities across the country. China has invested heavily in its key project “Stem Cell and Tissue Engineering for Regenerative Medicine”, and the Chinese Tissue Engineering Research and Development Centre has 14 pending patents. The Brazilian government, meanwhile, has invested $US4.3 million to conduct a three-year clinical trial of autologous stem cell therapy for the treatment of heart disease [7]. However, a recent study also identified regenerative medicine activities to varying degrees in 31 low- and middle-income countries, including, for example, Cuba, Argentina, South Africa, Egypt, Iran, and Malaysia [7].

The engagement in regenerative medicine by the developing world suggests that these countries themselves see a potential for this field to address local health needs. The differing levels of regenerative medicine activity among developing countries highlight an opportunity for South–South collaborations to address the emerging South–South divide between more-developed countries such as China, India, and Brazil, and the less-developed countries.

In terms of feasibility of implementation, it should be noted that almost all Asian, Latin American, and some African countries already carry out organ transplantation [21], and that certain regenerative medicine therapies, such as cell therapy, may be technically much simpler than traditional transplantation.

**The Six Criteria That Informed the Panellists' Choices**

**Burden:** Will the application address some of the most pressing health needs of developing countries?

**Impact:** Will the application be an improvement over currently existing treatment options and have a clear impact on health in developing countries?

**Feasibility:** Can the application reasonably be developed and deployed within a time frame of ten years?

**Affordability:** Will the application be affordable to develop and/or use in developing countries?

**Acceptability:** Is the application safe and socially, ethically, and legally acceptable?

**Indirect benefits:** Does the application provide benefits, such as capacity building or economic growth, which could indirectly improve health in developing countries?

## Implications and Recommendations

This is the first study to systematically identify and prioritize which applications of regenerative medicine are the most promising for improving health in developing countries. Technology foresight studies such as this have several benefits. They encourage long-term thinking, foster better coordination between stakeholders, encourage innovation by increasing communication, and help develop a shared future vision and commitment to specific goals [22]. In particular, these results may be used as a guide for the policy formulation of international and bilateral aid agencies, and by developing countries to help target policy aimed at initiating or developing further their existing capacity in regenerative medicine.

We propose several actions based on this research. First, we propose an initiative on grand challenges in noncommunicable diseases. This proposal is based on the successful experience of the Grand Challenges in Global Health initiative supported by the Bill and Melinda Gates Foundation, the Foundation for the National Institutes of Health, the Wellcome Trust, and the Canadian Institutes of Health Research [23], which was largely focused on infectious diseases. Our proposal would aim to address the key behavioural, scientific, and technological barriers in preventing and managing noncommunicable diseases. As highlighted by the results of this study, regenerative medicine could potentially play a key role in addressing some of the issues identified, though the exact extent of the contribution will depend both on the trajectory of research in the field and on the cost of emerging products and approaches. The establishment of such an initiative may encourage the regenerative medicine community to develop products and approaches that are applicable, affordable, and accessible to the developing world. This program would be open to researchers anywhere in the world, and would primarily fund projects and research areas relevant to developing countries that are not already receiving support.

Second, we encourage the governments of developing countries to evaluate the appropriateness of these technologies in their own national contexts and to investigate potential collaborations, both with industrialized and developing countries, as a means of building capacity in regenerative medicine. A study of successful health biotechnology sectors in developing countries found that a policy of encouraging collaborations and resource sharing was feasible and played an important role in the success of this sector in several countries [19].

Finally, we encourage the governments of industrialized countries to devote a portion of their research and development spending to challenges facing the developing world. Such a commitment from the governments of industrialized nations would provide the resources to pursue the proposed grand challenges in noncommunicable diseases initiative and would provide incentives to develop regenerative medicine therapies that are relevant, accessible, and affordable to the developing world.

## Supporting Information

Table S1 List of Panel Members (53 KB DOC).
