# Supplementary material for: Regenerative Medicine and the Developing World
Source: PLoS Med. 2006 Sep 12;3(9):e381. doi: 10.1371/journal.pmed.0030381 (PMC1564180; doi:10.1371/journal.pmed.0030381)
Supplement: Supplementary file 1 [file pmed.0030381.st001.doc]

Table S1. Panel Members

| **Name** | **Institutional Affiliation** |
| --- | --- |
| Sultan Abdulali | Mafraq Hospital (Abu Dhabi, United Arab Emirates) |
| Mohamed Abdullah | Aga Khan Hospital (Nairobi, Kenya) |
| Abdulla Al-Khader | Division of Nephrology, Hypertension & Renal Transplantation, King Abdulaziz Medical City (Riyadh, Saudi Arabia) |
| Pablo Argibay | Institute of Research & Technology, Hospital Italiano de Buenos Aires (Buenos Aires, Argentina) |
| Dorairajan Balasubramanian | L.V. Prasad Eye Institute (Hyderabad, India) |
| Robert Brown | Tissue Repair & Engineering Centre, University College London (Stanmore, United Kingdom) |
| Pablo A. Caviedes | Institute of Biomedical Sciences, University of Chile (Santiago, Chile) |
| Thomas M.S. Chang | Artificial Cells & Organs Research Centre, McGill University (Montréal, Canada) |
| Alan Colman | ES Cell International (Biopolis, Singapore) |
| Shimon Efrat | Department of Human Genetics & Molecular Medicine, Tel Aviv University (Tel Aviv, Israel) |
| Farha El-Chennawi | Immunology Unit, Mansoura University (Mansoura, Egypt) |
| Y. Murat Elçin | Tissue Engineering & Biomaterials Laboratory, Ankara University (Ankara, Turkey) |
| Dan Enescu | Plastic Surgery & Burns Department, Grigore Alexandrescu Hospital (Bucharest, Romania) |
| Marta R. Fontanilla | Molecular Biology Unit, Universidad El Bosque (Bogota, Colombia) |
| Xiaobing Fu | Key Research Laboratory of Wound Repair (Beijing, China) |
| Jorge Genovese^*^ | Thercell Argentina (Buenos Aires, Argentina) |
| María Beatriz Guglielmotti | Department of Oral Pathology, Buenos Aires University (Buenos Aires, Argentina) |
| Marisa Roma Herson | Plastic Surgery Research Laboratory, Universidade de Sao Paulo (Sao Paulo, Brazil) |
| Lynn L.H. Huang | Institute of Biotechnology, National Cheng Kung University (Tainan, Taiwan) |
| Kent Jonsson | Department of Surgery, University of Zimbabwe (Harare, Zimbabwe) |
| Renato A.K. Kalil | Instituto de Cardiologia do Rio Grande do Sul (Porto Alegre, Brazil) |
| Del Kahn | Division of General Surgery, University of Cape Town (Cape Town, South Africa) |
| Soo Hyun Kim | Biomaterials Research Center, Korea Institute of Science and Technology (Seoul, South Korea) |
| Il Woo Lee | Department of Neurosurgery, Daejon St. Mary’s Hospital (Daejon, South Korea) |
| Edison T. Liu | Genome Institute of Singapore (Biopolis, Singapore) |
| Mona K. Marei | Faculty of Dentistry Tissue Engineering Laboratories, Alexandria University (Alexandria, Egypt) |
| Betina Martínez^**^ | Pharmatrix Laboratories (Buenos Aires, Argentina) |
| Hamid Mirzadeh | Iran Polymer and Petrochemical Institute (Tehran, Iran) |
| Farhat Moazam | Sindh Institute of Urology and Transplantation (Karachi, Pakistan) |
| Nabil Mohsin | Department of Nephrology, Royal Hospital (Muscat, Oman) |
| Satdarshan P.S. Monga | McGowan Institute of Regenerative Medicine (Pittsburg, United States) |
| Rafique Moosa | Renal Unit, Tygerberg Hospital (Tygerberg, South Africa) |
| Marcelo Nacucchio^**^ | Pharmatrix Laboratories (Buenos Aires, Argentina) |
| Carola Niesler | Department of Physiological Sciences, University of Stellenbosch (Stellenbosch, South Africa) |
| Gustav Nossal | Department of Pathology, University of Melbourne (Victoria, Australia) |
| Xuetao Pei | Academy of Military Medical Sciences Stem Cell Research Center (Beijing, China) |
| Sheikh Riazuddin | National Centre of Excellence in Molecular Biology (Lahore, Pakistan) |
| Horacio L.R. Rilo | Cellular Transplantation Program, University of Cincinnati (Cincinnati, United States) |
| Ugo Ripamonti | Bone Research Laboratory (Parktown, South Africa) |
| Adib Rizvi | Sindh Institute of Urology and Transplantation (Karachi, Pakistan) |
| Faissal A.M. Shaheen | Saudi Center for Organ Transplantation (Riyadh, Saudi Arabia) |
| Duncan Stewart | Division of Cardiology, St. Michael’s Hospital (Toronto, Canada) |
| Cristina Velasquillo | Tissue Engineering & Cell Therapy Unit, National Center for Rehabilitation (Mexico City, Mexico) |
| Gordana Vunjak-Novakovic | Department of Biomedical Engineering, Columbia University (New York, United States) |
| Peter Zilla | Department of Cardiothoracic Surgery, University of Cape Town (Cape Town, South Africa) |

* During the course of this study, Jorge Genovese relocated to the McGowan Institute of Regenerative Medicine, Pittsburgh

^**^ Betina Martínez and Marcelo Nacucchio worked together and, hence, were counted as a single response for ranking purposes.
